# Loss of CFTR function in macrophages alters the cell transcriptional program and delays lung resolution of inflammation

**DOI:** 10.3389/fimmu.2023.1242381

**Published:** 2023-11-16

**Authors:** Dianne Wellems, Yawen Hu, Scott Jennings, Guoshun Wang

**Affiliations:** Department of Microbiology, Immunology and Parasitology, Louisiana State University Health Sciences Center, New Orleans, LA, United States

**Keywords:** cystic fibrosis, CFTR, macrophage-specific CF mouse model, lung infection and inflammation, single-cell RNA sequencing

## Abstract

Cystic fibrosis (CF) is an autosomal recessive genetic disorder caused by mutations in the CF Transmembrane-conductance Regulator (CFTR) gene. The most severe pathologies of CF occur in the lung, manifesting as chronic bacterial infection, persistent neutrophilic inflammation, and mucopurulent airway obstruction. Despite increasing knowledge of the CF primary defect and the resulting clinical sequelae, the relationship between the CFTR loss of function and the neutrophilic inflammation remains incompletely understood. Here, we report that loss of CFTR function in macrophages causes extended lung inflammation. After intratracheal inoculation with *Pseudomonas aeruginosa*, mice with a macrophage-specific *Cftr*-knockout (Mac-CF) were able to mount an effective host defense to clear the bacterial infection. However, three days post-inoculation, Mac-CF lungs demonstrated significantly more neutrophil infiltration and higher levels of inflammatory cytokines, suggesting that Mac-CF mice had a slower resolution of inflammation. Single-cell RNA sequencing revealed that absence of CFTR in the macrophages altered the cell transcriptional program, affecting the cell inflammatory and immune responses, antioxidant system, and mitochondrial respiration. Thus, loss of CFTR function in macrophages influences cell homeostasis, leading to a dysregulated cellular response to infection that may exacerbate CF lung disease.

## 1 Introduction

The cystic fibrosis transmembrane-conductance regulator gene (*CFTR)* encodes a cAMP-activated chloride and bicarbonate channel (1–4). Its dysfunction leads to clinical cystic fibrosis (CF), an autosomal recessive genetic disorder that affects 1/~3000 live births in the US and ~100,000 individuals worldwide (5). CF is a systemic disease that affects many organs and systems, including the lung, gastrointestinal tract, pancreas, hepatobiliary system, reproductive system, and sweat gland. However, most life-threatening complications occur in the lung and present as chronic bacterial infections, persistent neutrophilic inflammation, and small airway obstruction (6, 7). Strikingly, 60 to 80% of CF lungs are colonized by two opportunistic bacterial pathogens, *Pseudomonas aeruginosa* (PsA) and *Staphylococcus aureus* (8, 9). *Staphylococcus aureus* is often one of the first bacteria detected in the sputum of children; however, by around 24-35 years of age, PsA becomes the dominant bacterial pathogen (9–11). Unfortunately, PsA in the sputum has been associated with a decline in lung function and a worse prognosis (12–14). Previous studies have demonstrated that the absence of functional CFTR in airway epithelial cells compromises lung mucociliary clearance, resulting in an inability to clear bacteria [Reviewed in (15)]. As the host lung defense against infection requires a collaborative action between intrinsic pulmonary elements and mobilized immune factors, how the CF immune system contributes to the CF lung disease has not been completely defined.

Upon microbial challenge, a healthy lung effectively mobilizes immune cells for defense. Lung-resident alveolar macrophages (AM) are yolk-sac-derived, populate during embryonic hematopoiesis, and serve as the sentinel cells to rally marrow-derived immune cells through the secretion of pro-inflammatory cytokines/chemokines (16). Neutrophils are the first recruited immune cells tasked with containment and degradation of the invading microbes. Following neutrophils, circulating monocytes arrive at the lung and differentiate into monocyte-derived macrophages (MDM) to aid the anti-microbial battle and to clear consumed neutrophils and other apoptotic cells through efferocytosis (17). CFTR dysfunction affects these innate immune cells. Neutrophils from CF patients are deficient in phagosomal chloride transport and hypochlorous acid production, which leads to defective microbial killing (18–20), among other abnormalities [Reviewed in (21)]. Macrophages from CF patients have also shown intrinsic defects in metabolism, efferocytosis, inflammatory pathways, and response to pathogens (22–26).

Using a pan-*Cftr*-knockout (KO) mouse model, Bruscia et al. demonstrated that CF lungs are hyper-inflammatory after LPS challenge. Additionally, CF macrophages, isolated from the challenged lungs or derived from the bone marrow, produce significantly more inflammatory cytokines (27, 28). Bonfield and colleagues documented that myeloid lineage-specific *Cftr*-knockout (KO) mice have a slower resolution of inflammation and infection (29). Our previous investigations confirmed these findings, demonstrating that CFTR loss of function in neutrophils and monocytes/macrophages leads to a defective lung bacterial clearance (30) and prolonged neutrophilic inflammation (31). As neutrophils and macrophages have many overlapping functions, the distinct role of macrophages in the compromised lung defense has yet to be clearly delineated.

In the current study, we generated a novel macrophage-specific *Cftr*-KO (Mac-CF) mouse model. The Mac-CF mice and their congenic control (Ctrl) were intratracheally challenged with PsA, and their lung responses were examined and compared. Single-cell RNA sequencing (scRNA-seq) was performed to reveal potential differences in immune landscaping in the Mac-CF and Ctrl lungs. Gene signatures of Mac-CF and Ctrl macrophages in the bacteria-challenged lungs were also identified. The obtained data suggest that CFTR loss of function in macrophages does not affect lung bacterial clearance but affects lung resolution of inflammation. This dysregulated response by the CF macrophages was found to be associated with alterations in their transcriptional program.

## 2 Materials and methods

### 2.1 Reagent and resource availability

This study generated a macrophage-specific CF mouse model, which will be available to the research community under a standard inter-institutional transfer agreement. Reagent and resource information is provided in **Supplementary Table 1**. Inquiries and requests should be directed to the lead correspondent, Guoshun Wang (gwang@lsuhsc.edu).

### 2.2 Animals and ethics of animal use

The macrophage-specific *Cftr*-KO (Mac-CF) mouse line was established by breeding *Cftr-*exon-10-floxed (*Cftr* ^fl10/fl10^) mice (32) with Cx3cr1^cre/+^ mice (The Jackson laboratory) (33), as outlined in the schematic (**Supplementary Figure 1A**). Mac-CF (*Cftr* ^fl10/fl10^ *Cx3cr1* ^cre/+^) and the sibling congenic control (Ctrl) (*Cftr* ^fl10/fl10^ *Cx3cr1* ^+/+^) mice were screened by PCR using the genotyping primers (**Table 1**). All animals used in the study were age and sex-matched, ranging from 6-12 weeks old. The animals were handled according to the ethical standards set by the Guide for Care and Use of Laboratory Animals of the National Institutes of Health, and approved by the Louisiana State University Health Sciences Center Animal Care and Use Committee.

**Table 1:** PCR primers for validation of CFTR knockout.

Mouse CFTR Genotyping Primers		
	Bases	Sequence (5’ to 3’)
Primer 1	20	GTAGGGGCTCGCTCTTCTTT
Primer 2	20	GTACCCGGCATAATCCAAGA
Primer 3	20	AGCCCCTCGAGGGACCTAAT
Mouse CRE Genotyping Primers		
	Bases	Sequence (5’ to 3’)
Cre-112 F	20	GTCCGTTTGCCGGTCGTCGG
Cre-112 R	20	CAGACCGCGCGCCTGAGGAT

### 2.3 Validation of Mac-CF mouse model

To validate macrophage-specific *Cftr*-exon-10 deletion, 1 ml of a sterile 9% casein PBS solution was injected into the intraperitoneal cavity (i.p.) of Mac-CF mice, followed by peritoneal lavage two days later. The obtained cells were blocked with TruStain FcX™ PLUS (anti-mouse CD16/32) (Biolegend Cat#156604) antibody, and immunostained with either FITC anti-mouse F4/80 antibody (BioLegend; clone BM8, Cat #123108) or FITC IgG Rat Isotype control antibody (Invitrogen eBiosciences Cat#11-4321-80). F4/80-positive and -negative cells were, separately, sorted out by FACS AriaII (BD Biosciences) at LSUHSC Flow Cytometry Core and genotyped by PCR (**Supplementary Figure 1B**).

### 2.4 Lung bacterial challenge, bronchoalveolar lavage fluid (BALF) collection and tests

*Pseudomonas aeruginosa* (PsA), a clinical isolate with the mucoidal phenotype, were grown aerobically (1:10 media: flask ratio) in Tryptic Soy Broth (TSB) (Sigma; Cat# 22092) for 9 hours at 225 rpm at 37°C. Unless otherwise stated, mice were intratracheally inoculated with 2.0x10^7^ CFU of PsA in 50 μl of PBS. At specified time points (Day 0, 2, 3, or 5 post-inoculation), bronchoalveolar lavage was performed as previously described (30, 31). BALF of each assigned animal was obtained for bacterial quantification, total cell counting, cell differential counting, and inflammatory cytokine measurements. For bacterial survival assessment, each BALF sample was appropriately diluted and plated on tryptic soy agar. Colonies were enumerated after overnight culture at 37°C to calculate the total CFU. Total BALF cell counts were obtained by a Cellometer K2 (Nexcelom) with AOPI staining (Nexcelom, Cat# CS2-0106). For cell differential counting, BALF cells were cytospun onto a microscopic slide, fixed, and stained with the Harleco Hemacolor Stain Set (Sigma; Cat #65044-93). The slides were examined by microscopy. At least 200 nucleated cells in randomly selected views of each slide were counted for neutrophils, macrophages, and lymphocytes. For cytokine measurements, IL-6, TNF-α, MIP-2, IL-1β, and KC were quantified using DuoSet Mouse ELISA kits (R&D Biosystems, BioTechne Cat# DY-406, 410, 452, 401, 453, respectively) according to the manufacturer’s recommendations.

### 2.5 Lung histology

Mouse lungs assigned for histology were isolated and inflated with 1 ml of 4% paraformaldehyde in PBS for fixation. The fixed lung tissues were sent for paraffin embedding, slicing, and H&E staining at the LSUHSC Cell Morphology Core. Slides were then examined microscopically.

### 2.6 Differentiation, confirmation, and LPS-stimulation of bone marrow-derived macrophages (BMDM)

According to the published protocol (34–37), mouse bone marrows were extracted from femur and tibia bones. After passing through a 70-micron strainer, the cells were re-suspended in DMEM (Gibco, Cat#11965-092) supplemented with 10% FBS, 1% antibiotic-antimycotic (Gibco; Cat #15240096), 5 mM GlutaMAX (ThermoFisher; Cat#35050061), and 20% of the conditioned media from L929 cells (ATCC RRID: CVCL_0462). Then, the bone marrow cells were seeded in non-tissue culture petri dishes at approximately 2-3.0x10^5^ cells per ml and cultured for seven days with a media change three days after the initial seeding. To confirm macrophage differentiation, the cells were dissociated by trypsinization and stained with FITC anti-mouse F4/80 antibody, as described above, for flow cytometry. Cell viability, measured via AO/PI staining and cellometer counting, was found to be greater than 94%. To assess cell response to LPS stimulation, BMDM (1x10^5^ live cells) from Mac-CF and Ctrl mice were exposed to 16 μg/ml PsA LPS (Sigma; Cat#L9143) in complete DMEM with 10% heat-inactivated human AB serum (HS) (Sigma; Cat#HS3667). The supernatant was collected at 2, 4, and 8 hours post-LPS stimulation. Cytokine/chemokine levels were assessed by ELISA as described above.

### 2.7 *In vitro* bacterial killing by BMDM

For *in vitro* bacterial killing, BMDM were incubated in Trypsin-EDTA (0.25%) solution (ThermoFisher; Cat# 25200056) to dislodge them from the petri dish. Next, the cells were washed twice with DMEM without any supplements. Then, 2.5x10^5^ BMDM were incubated with human AB serum-opsonized PsA at an MOI of 6 for 30 minutes at 37°C in an end-over-end rotator. To establish the initial phagocytosis, 1/5 of the incubated mixture was sampled and placed in complete DMEM with gentamicin (100 µg/ml) (Sigma Cat# G1914) and left on ice. To the remaining mixture, gentamicin was added, and incubated for another 30 minutes at 37°C in an end-over-end rotator. The non-phagocytosed bacteria and gentamicin were removed by centrifuging (250x *g*, 4°C, 5 minutes) and the pelleted BMDM were washed with PBS before being suspended in complete DMEM. BMDM from the Ctrl and Mac-CF samples were aliquoted into a 24-well cell culture dish and cultured for 1, 3, 10, and 22 hours. At the indicated time, wells were washed with PBS, and cells were dislodged using trypsin and mechanical force and suspended in saponin (5mg/ml) water solution for 10 minutes. BMDM were further lysed by vortexing and centrifuging at 12,000x g for 15 minutes at 4°C. The supernatants were serially diluted, plated onto tryptic soy agar, and cultured overnight at 37°C. Colonies were counted to establish CFU per ml.

### 2.8 ScRNA sequencing – sample collection, cell hashing, library construction and sequencing

Mac-CF and Ctrl mice were intratracheally inoculated with 2.0x10^7^ CFU of PsA in PBS. BAL cells were collected from each mouse at Days 1, 3 and 5 post-inoculation. The same time-point samples from 4 mice of each genotype were equally pooled and submitted for scRNA-seq. Five thousand BAL cells from each genotype per time point were acquired for scRNA-seq using the 10X Genomics platform.

### 2.9 ScRNA-seq data and processing

The primary analyses were performed using 10x Cellranger V.6.1.2, aligning the sequencing reads to the mm10, the mouse transcriptome, and quantification of transcript expressions in each cell. The output files with barcodes, features, and matrix were used for downstream quality control and data analyses, using R programming 4.2.2 and Seurat package version 4.3.0, except where otherwise noted (38–43).

The individual files of Ctrl and Mac-CF data were loaded using the Read10X() function. For quality control, cells containing less than 5% mitochondrial counts and cells having unique features of less than 200 and more than 6000 (nFeature_RNA > 200 & nFeature_RNA < 6000 & percent.mt < 5.0), along with negative and doublet cells were removed from downstream analysis to ensure quality reads. The remaining cells were demultiplexed with HTO_classification, normalized, and scaled using functions: NormalizeDate(), and SCTransform() with method “glmGamPoi” (44, 45).

After quality control, 24,149 high-quality cells were deemed for downstream processing, including 4,752 cells from Day 1_Ctrl group, 4,195 cells from Day 3_Ctrl group, 4,084 cells from Day 5_Ctrl group, 3,961 cells from Day 1_Mac-CF group, 3,687 cells from Day 3_Mac-CF group, and 3,470 cells from Day 5_Mac-CF group. Then, the Ctrl and Mac-CF data sets were integrated via Harmony package version 0.1.1 (46).

### 2.10 Unsupervised clustering and cell annotation

The integrated dataset was normalized and scaled as described above. The first 30 principal components (PCs) of the integrated dataset were used to compute a Uniform Manifold Approximation and Projection (UMAP) of the cells. The annotation of each cell type was identified against an established mouse immune cell database (ImmGen) by using the package SingleR version 1.10.0 (47, 48), and confirmed by well-known immune cells markers. Notably, *S100a8* and *S100a9* are the markers for neutrophils. *Cd68* is a marker for macrophages and monocytes. *Itgax* and *Siglecf* are the markers for AM. *Ly6c2* is a monocyte marker and expressed in monocyte-derived macrophages. *Cd3e* and *Cd3g* are the markers for T lymphocytes (48–52).

### 2.11 Identification of differentially epressed genes (DEGs) and analysis of gene ontology (GO)

The identification of DEGs was accomplished using the FindMarkers() or FindAllMarkers() function, with a minimum percentage of 0.25, and log_2_ fold-change of 0.5 (logfc.threshold=0.5). The DEGs (p.adj < 0.05, log_2_FC > 0.5) were further separated into upregulated and downregulated genes in the Mac-CF population on days post-inoculation (**Supplementary Data 1** and **2**). Using the R package ClusterProfiler version 4.6.0, the DEGs were enriched and mapped to the Gene Ontology (GO) against the mouse database (org.Mm.eg.db), with a p-adjusted value of less than 0.05 by Bonferroni correction (53–55).

### 2.12 Scoring of biological function

Individual cells were scored for their expression of gene signatures representing certain biological functions (**Supplementary Data 3**). Each functional signature gene set was derived from the Molecular Signatures Database (MSigDB) (56–59), including NADH dehydrogenase activity (GO:0003954), NADH dehydrogenase (quinone) activity (GO:0050136), Glutathione metabolic processes (GO:0006749), Glutathione peroxidase activity (GO:0004602), Glutathione transferase activity (GO:0004364), NRLP1 inflammasome complex (GO:0072558), Positive regulation of IL-1 production (GO:0032732), Superoxide dismutase activity (GO:0004784), and Cellular response to reactive oxygen species (ROS) (GO:0000302). Each signature functional scores reflecting certain gene set activity were calculated by R package: AUCell 1.18.1 (60).

### 2.13 Statistics

Statistical significance was determined using Student’s t-test or two-way ANOVA test with Tukey test. Statistical significance was determined using GraphPad 9 or R programming.

## 3 Results

### 3.1 Macrophage-specific CF mouse model

The Mac-CF mouse line was generated by breeding *Cftr-*exon-10-floxed (*Cftr* ^fl10/fl10^) mice with Cx3cr1^Cre/+^ mice, as depicted (**Supplementary Figure 1A**). To confirm macrophage-specific *Cftr-*exon-10 deletion, peritoneal inflammatory cells from Mac-CF mice, induced by a casein solution injection, were harvested, stained with an F4/80 antibody, and FACS-sorted. PCR genotyping of the sorted cells with the specific primers (**Table 1**) demonstrated that the F4/80-positive cells, but not the negative ones, had specific *Cftr*-exon-10 deletion (**Supplementary Figure 1B**), indicating that the Mac-CF mice had the designed *Cftr*-exon-10 deletion in macrophages alone. Moreover, macrophages, differentiated *in vitro* from Mac-CF and Ctrl mouse bone marrow, were also genotyped by PCR, confirming that *Cftr*-exon-10 deletion only occurred in Mac-CF macrophages (**Supplementary Figure 1C**).

### 3.2 Loss of CFTR in macrophages prolongs lung inflammation after bacterial challenge

**Figure 1:**
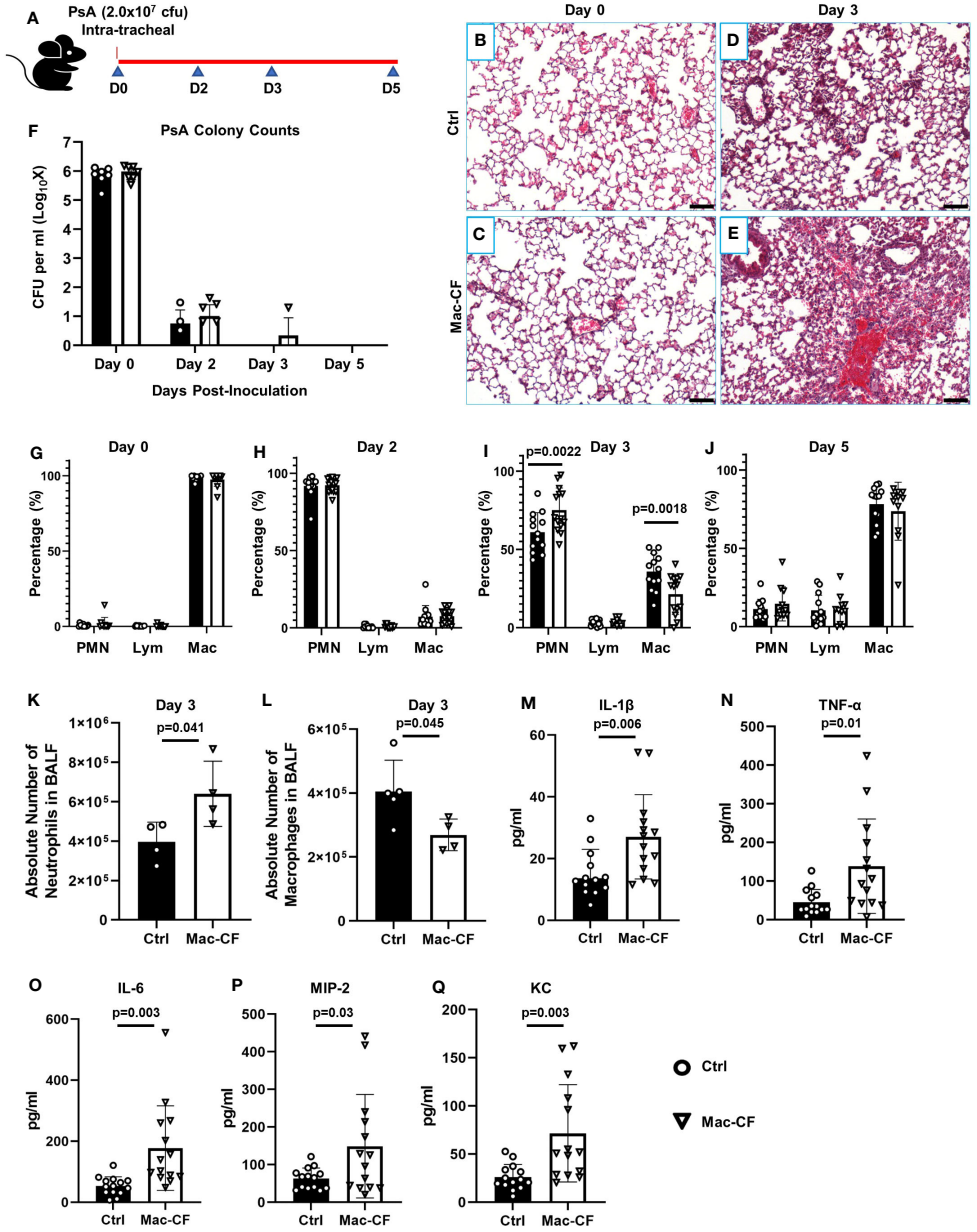
Loss of CFTR in macrophages prolongs lung inflammation after PsA challenge. **(A)** Schematic of experimental design. Mouse lungs, intratracheally challenged with 2.0x10^7^ CFU PsA, were subjected to histological examination and bronchoalveolar lavage at Days 0, 2, 3, and 5 post-inoculation. **(B–E)** Representative H&E images of the lungs from Ctrl and Mac-CF mice at Day 0 and 3 after PsA inoculation. Scale bar (100 μm). **(F)** Lung clearance of PsA. **(G–J)** Cell differential counting of BALF cells from Ctrl and Mac-CF mice at 4 time points. Data were from three independent experiments with a combined total of 12-15 mice per genotype and per time point. Significant differences were only found at the time point of Day 3 post-challenge. Statistical differences were determined by Two-way ANOVA with Tukey test. **(K–L)** Absolute cell number in BALF at Day 3 post-challenge from a representative experiment. **(K)** Total neutrophil number. **(L)** Total macrophage number. Statistical differences were determined by Student’s t-test (n=4-5, p<0.05). **(M–Q)** Quantification of pro-inflammatory cytokines and chemokines in BALF from Day 3 post-challenge by ELISA. Data were from three independent experiments with a combined total of 12-15 mice per genotype and per time point. Statistical differences were determined by Student’s t-test.

To assess how CFTR loss of function in macrophages affects lung defense against bacterial infection, we intratracheally challenged the Mac-CF and Ctrl mice with PsA (2.0x10^7^ CFU). At the outlined time points (**Figure 1A**), assigned animals were sacrificed to assess lung infection and inflammation. Histological examination showed that immediately after bacterial inoculation (Day 0), lungs from both Mac-CF and Ctrl mice had a similar cell density and structural appearance (**Figures 1B, C**). However, three days post-inoculation, the Mac-CF lung demonstrated greater inflammatory cell infiltration (**Figures 1D, E**). To quantify lung infection, bronchoalveolar lavage fluid (BALF) samples were plated on tryptic soy agar and cultured overnight. Colony enumeration revealed that PsA in both Ctrl and Mac-CF lungs were substantially reduced by Day 2, nearly cleared by Day 3, and undetectable by Day 5 post-inoculation. No significant differences were found between the two genotypes at any given time point (**Figure 1F**).

We next performed total cell number and cell differential countings of each BALF sample to quantify lung inflammation. Immediately after bacterial inoculation (Day 0), the predominant cells in both Mac-CF and Ctrl lungs were macrophages (**Figure 1G**). However, Day 2 post-inoculation neutrophils became the dominant cell type, accounting for over 90% of the BALF cells (**Figure 1H**). Day 3 post-inoculation Ctrl lungs began to resolve the inflammation, exemplified by a significantly greater percentage of macrophages and a significantly lower percentage of neutrophils. In contrast, Mac-CF lungs in this time remained neutrophil-predominant, indicating a state of prolonged neutrophilic inflammation (**Figure 1I**). The absolute number of neutrophils and macrophages in Mac-CF lungs at Day 3 also confirmed the extended neutrophilic state (**Figures 1K, L**).

We further evaluated if the prolonged neutrophilic inflammation in Mac-CF lungs had any correlation with increases in proinflammatory cytokines/chemokines. The levels of IL-1β, TNF-α, IL-6, MIP-2, and KC in the BALF were measured by ELISA. None of these cytokines/chemokines had any significant difference between the genotypes at all time-points except Day 3 (**Figures 1M–Q**). Mac-CF lungs had significantly higher levels of IL-1β, TNF-α, IL-6, MIP-2 and KC than the Ctrl lungs.

Taken together, the data indicate that Mac-CF lungs were capable of mounting an effective anti-bacterial response. Despite no difference in bacterial clearance, 3 days post-inoculation, Mac-CF lungs had significantly more neutrophil infiltration and cytokine/chemokine production. This suggests that loss of CFTR function in macrophages resulted in a prolonged inflammatory response to the bacterial challenge.

### 3.3 Bone marrow-differentiated macrophages (BMDM) from Mac-CF mice produce more cytokines/chemokines and exhibit decreased bactericidal ability

We posited that the excessive cytokines/chemokines observed in the Mac-CF lungs might be contributed by CF macrophages. To test this, bone marrow cells from Ctrl and Mac-CF mice were harvested and differentiated into respective mature macrophages, as outlined (**Supplementary Figure 2A**). F4/80 antibody staining phenotypically confirmed the differentiation (**Supplementary Figure 2B**). Then, the obtained BMDM were stimulated with PsA-LPS for 2, 4, and 8 hours, and cytokine/chemokine levels in the culture media were measured. While both Ctrl and Mac-CF macrophages had a time-dependent increase in cytokine/chemokine production, the Mac-CF cells secreted significantly more IL-6, MIP-2, and TNF-α at 8 hours post-LPS stimulation (**Supplementary Figures 2C–F**). Thus, we conclude that *Cftr-*exon-10 deletion in macrophages rendered the cell hyper-responsive to stimulation, producing significantly more proinflammatory cytokines/chemokines.

The *in vivo* observation that Mac-CF and Ctrl lungs had a comparable bacterial load prompted us to compare the bacterial killing capacity of Mac-CF and Ctrl macrophages *in vitro*. The BMDM derived from Mac-CF and Ctrl mice were exposed to human AB serum-opsonized PsA at a multiplicity of infection (MOI) of 6. Rates of phagocytosis were determined 30 minutes after the exposure, showing no significant difference between the two genotypes (**Supplementary Figure 2G**). However, Mac-CF BMDM had a significantly higher intracellular bacterial burden 22 hours post-PsA exposure than the Ctrl counterparts (**Supplementary Figure 2H**), indicating a bacterial killing defect in the CF macrophages *in vitro*.

### 3.4 Loss of CFTR in macrophages alters the lung immune cell landscape upon bacterial challenge

**Figure 2:**
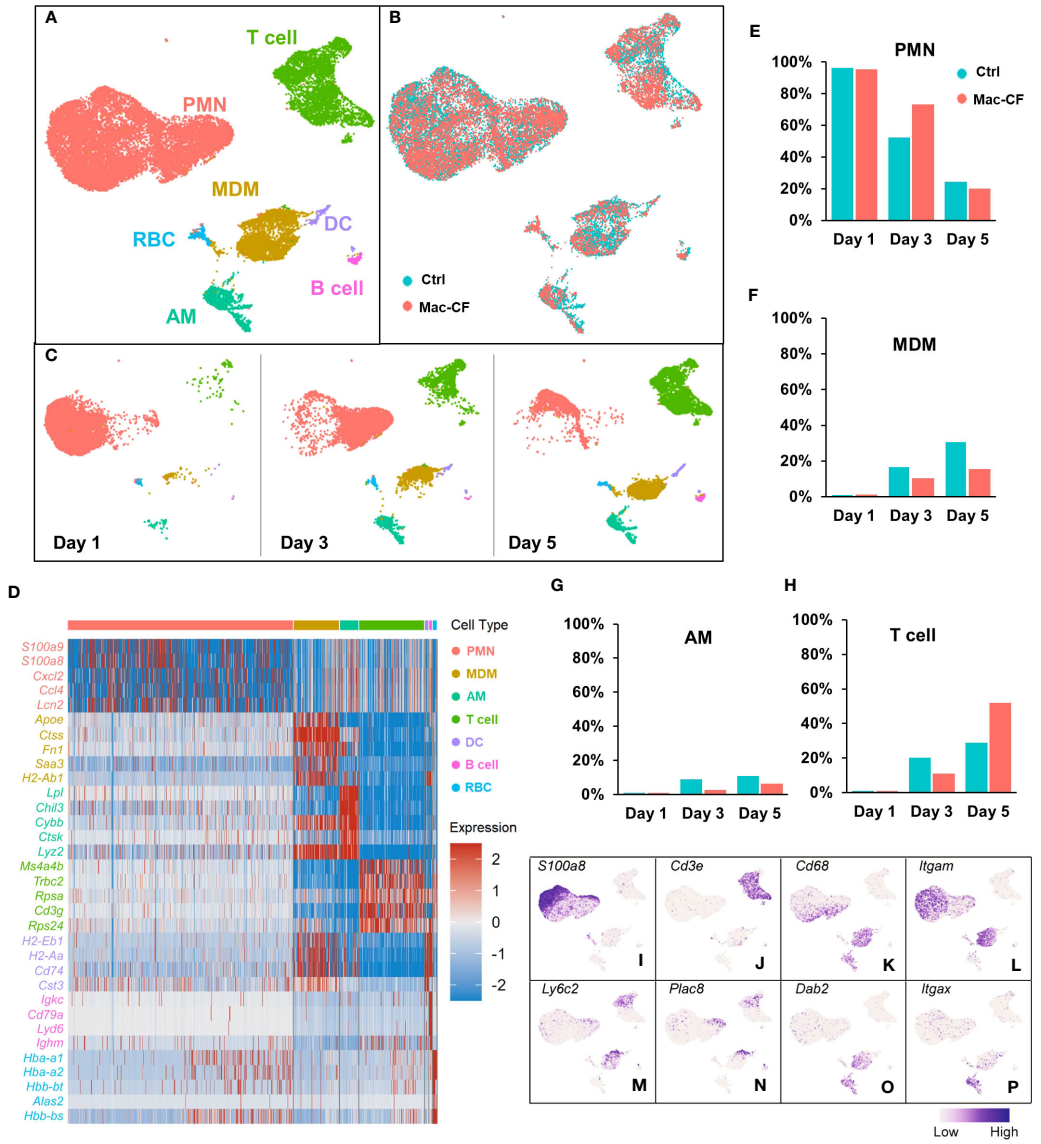
Atlas of the BAL cells from Ctrl and Mac-CF mice. **(A–C)** Cell clustering of BAL cells from Ctrl and Mac-CF mice via Uniform Manifold Approximation and Projection (UMAP) by: **(A)** Cell types; **(B)** Genotypes; **(C)** Time points. **(D)** Heatmap of top 5 differentially expressed genes (DEGs) within each cell type. **(E–H)** Percentages of major cell types in BALF from each genotype at different time points: **(E)** Neutrophils (PMN); **(F)** Monocyte derived macrophages (MDM); **(G)** Alveolar macrophages (AM); **(H)** T cells. **(I–P)** Feature plots of cell specific marker genes in the BAL cells.

ScRNA-seq was employed to cluster the BALF immune cells from Ctrl and Mac-CF mouse lungs at Day 1, 3, and 5 post-PsA inoculation. The obtained transcripts were aligned with an established mouse immune cell database published by the Immunological Genome Project (ImmGen) (47, 48). Uniform Manifold Approximation and Projection (UMAP) of all BALF samples revealed seven distinct clusters of cells (**Figure 2A**): neutrophils (PMN), monocyte-derived macrophages (MDM), alveolar macrophages (AM), T lymphocytes (T cell), dendritic cells (DC), B lymphocytes (B cell), and red blood cells (RBC). Moreover, UMAP plotted by genotypes demonstrated an overlapping projection pattern (**Figure 2B**), and UMAP by time points showed dynamic changes of the immune cells in the lungs. PMN decreased with time, while MDM, AM, and T cells increased (**Figure 2C**). Heatmap shows the top 5 differentially expressed genes (DEGs) of each cell type (**Figure 2D**), representing their signature genes. Moreover, Day 1 post-inoculation over 90% of the BALF cells were PMN in both genotypes (**Figure 2E**). At Day 3 post-inoculation neutrophils reduced to ~50% in Ctrl BALF and ~75% in Mac-CF BALF, and on Day 5 post-inoculation the percentages of neutrophils were further lowered to ~20% for both genotypes (**Figure 2E**). These single cell-clustering data confirmed the cell differential counting data obtained by conventional microscopic assessment (**Figures 1I, J**).

Macrophages (MDM and AM) also exhibited different dynamics between the genotypes. Day 1 post-inoculation, AM and MDM were low (~1%) in both Ctrl and Mac-CF BALF (**Figures 2F, G**). However, Day 3 post-inoculation, the composition of MDM in Ctrl and Mac-CF BALF increased to ~17% and ~10%, respectively, and AM to ~9% and ~3% (**Figures 2F, G** **).** At Day 5 post-inoculation, MDM in Ctrl BALF accounted for ~31% and only ~16% in Mac-CF. AM in Ctrl BALF comprised ~11% and merely ~6% in Mac-CF (**Figures 2F, G**). Both MDM and AM had a slower increase in Mac-CF lungs than in Ctrl lungs. Interestingly, the percentage of T cells had markedly increased in the Mac-CF Day 5 post-inoculation (**Figure 2H**).

Feature plots marked the expression of signature marker genes for each type of immune cell. As expected, *S100a8* was predominantly expressed in the PMN population (**Figure 2I**), and *Cd3e* predominantly in the T cell population (**Figure 2J**). Previous publications have defined classical monocytes as *Cd68* ^+^ *Cd11b (Itgam)* ^+^ *Ly6c* ^+^ *Plac8* ^+^ *Dab2* ^-^ and mature macrophages as *Cd68* ^+^ *Cd11b* ^+^ *Ly6c* ^-^ *Plac8* ^-^ *Dab2* ^+^ (61–65). Intriguingly, we observed that *Plac8* and *Ly6c2* expressions were largely restricted to the upper area of the plotted MDM population (**Figures 2M, N**), while *Dab2* expression was mainly limited to the lower area of the same population (**Figure 2O**). Thus, MDM in the bacteria-challenged lungs had a progressive maturation from *Ly6c* ^+^ *Plac8* ^+^ *Dab2* ^-^ (a more monocyte-like MDM) to *Ly6c* ^-^ *Plac8* ^-^ *Dab2* ^+^ (a fully mature MDM). Moreover, the AM population displayed *Cd68^+^Cd11b(Itgam)^-^Cd11c(Itgax)^+^Dab2^+^* (**Figures 2K, L, O, P**), as previously published (61, 63–66). These signature genes, tabulated in **Table 2**, will be used to discern MDM from AM for further analyses.

**Table 2:** Gene markers for macrophage identification.

Cell Type	Expression Markers	Reference
Immature monocyte-derived macrophages	*Cd68^+^Cd11b(Itgam)^+^Ly6c^+^Plac8^+^Dab2^-^*	(61–65)
Mature monocyte-derived macrophage	*Cd68^+^Cd11b(Itgam)^+^Ly6c^-^Plac8^-^ Dab2^+^*	
Alveolar macrophage	*Cd68^+^Cd11b^-^Cd11c(Itgax)^+^Dab2^+^*	(61, 63–66)

### 3.5 CFTR loss of function in macrophages alters the cell transcriptional program

**Figure 3:**
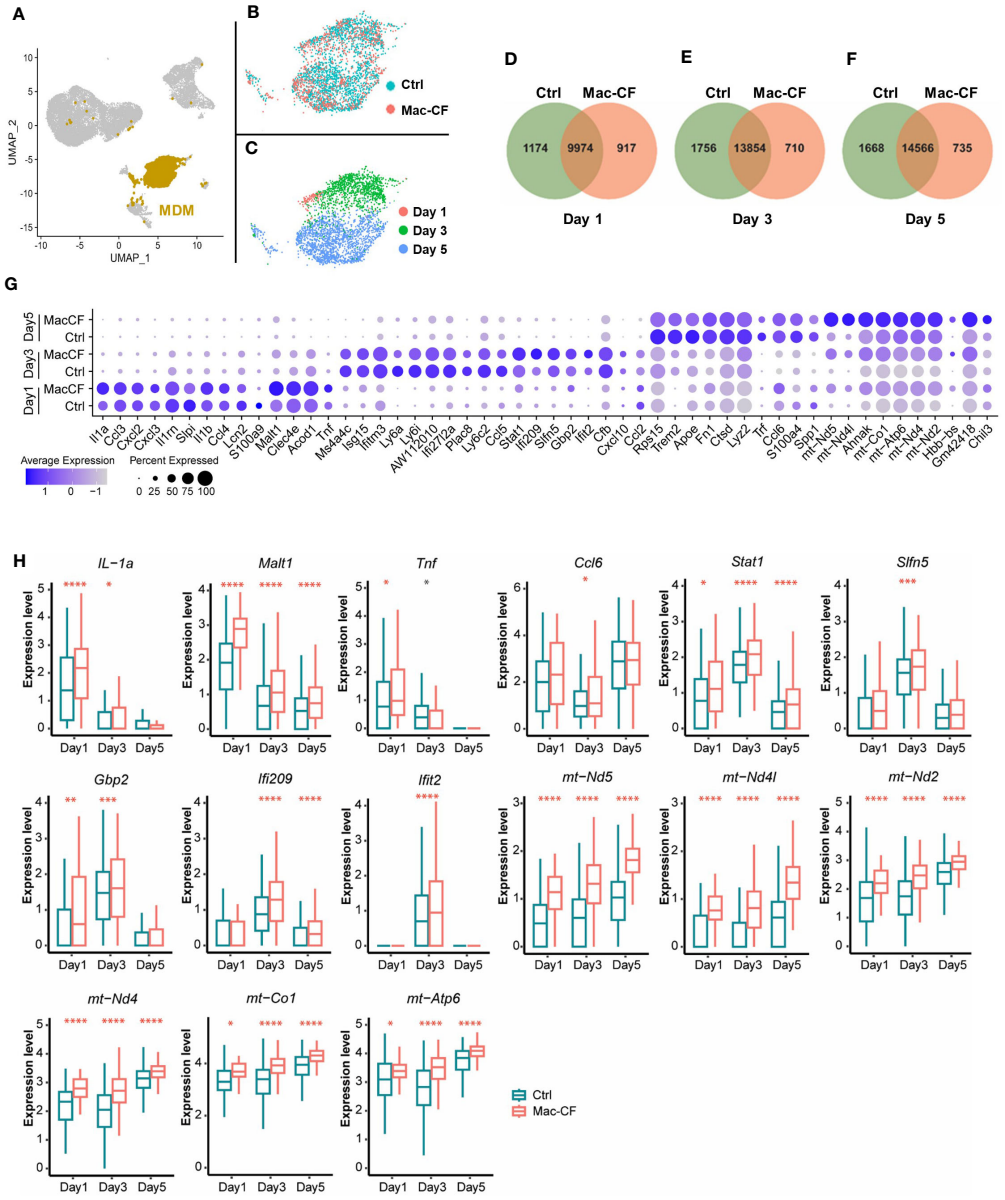
CF monocyte-derived macrophages (MDM) exhibit upregulation in mitochondrial associated gene expression. **(A–C)** Distribution of MDM in the BALF of Ctrl and Mac-CF Mice. UMAP illustrating MDM clustering **(A)** in the combined BALF cells, **(B)** by genotypes, or **(C)** by time points. **(D–F)** Venn diagram of unique and overlapping genes (average gene counts >1) between Ctrl and Mac-CF MDM. **(G)** Dot plot of top 10 DEGs in the Ctrl and Mac-CF MDM at 3 time points. **(H)** Box plots of representative DEGs in Mac-CF MDM. Each p-value was calculated using two-way ANOVA with Tukey test for multiple comparison (*p<0.05, **p<0.01, ***p<0.001, ****p<0.0001). Red stars indicate that the gene is expressed significantly higher in Mac-CF groups compared to Ctrl groups, while black stars indicate the opposite.

As *Cftr*-exon-10 deletion in our Mac-CF mice was only limited to the macrophage lineages, we thus focused our following analyses on MDM and AM. Cell clustering analysis indicated that MDM was projected as a unique population in the general UMAP (**Figure 3A**). We then extracted the MDM data and plotted them in separate UMAP by either genotypes (**Figure 3B**) or time points (**Figure 3C**). Projection patterns by genotypes overlapped; however, when plotted by days post-inoculation, the UMAP revealed distinct projection patterns. Venn diagrams depicted the common and unique genes of Ctrl and Mac-CF MDM at different time points. Despite sharing most of the expressing genes, the two genotypes had 5-10% of gene expressions unique to each genotype (**Figures 3D-F**). Top 10 DEGs of Ctrl and Mac-CF MDM at each time point were plotted in dot plot (**Figure 3G**). When compared to Ctrl MDM, the most significantly upregulated genes in Mac-CF MDM at one or more time points were found to relate to 1) inflammatory cytokines/chemokines (*IL-1α*, *Tnf*, and *Ccl6*), 2) immunity (*Malt1*, *Stat1*, *Ifi209*, *Slfn5*, *Gbp2*, and *Ifit2*), and 3) mitochondrial oxidative phosphorylation (*mt-ND2, 4, 4L, 5, mt-Co1* and *mt-Atp6*) (**Figure 3H**). Notably, the mitochondrial genes in Mac-CF MDM were upregulated from Day 1 post-PsA challenge and remained higher than Ctrl MDM on the later time points post-inoculation (**Figures 3G, H**).

### 3.6 MDM in Mac-CF lungs have altered biological processes and molecular functions in mitochondria

**Figure 4:**
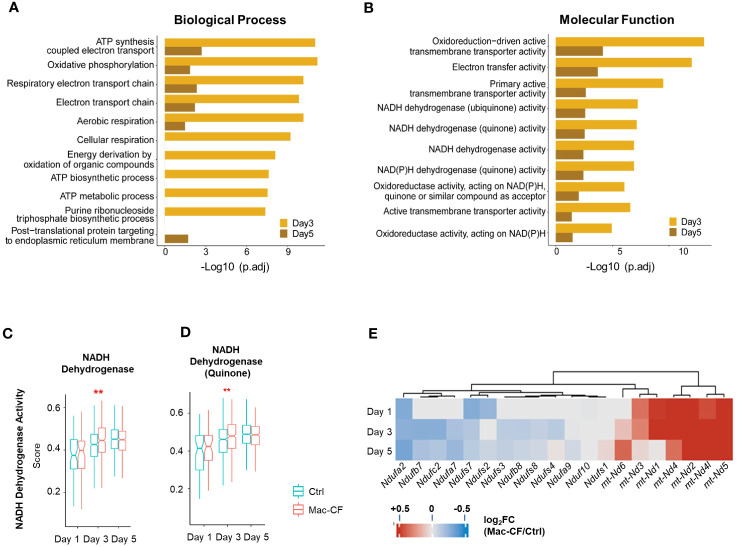
Monocyte derived macrophages (MDM) have increased mitochondrial respiration gene activities. **(A, B)** Gene ontology (GO) enrichment of DEGs in Mac-CF MDM as compared to Ctrl MDM at 3 and 5 days post-inoculation. **(A)** Top 10 enriched GO biological process terms; **(B)** Top 10 GO molecular function terms. **(C, D)** Scores of the NADH dehydrogenase activity. **(C)** NADH dehydrogenase activity (GO:0003954); **(D)** NADH dehydrogenase (Quinone) activity (GO:0050136). **(E)** Heatmap showing the differences of log_2_[fold-change] in the expression of mitochondrial genes between Mac-CF and Ctrl MDM. Each p-value was calculated using two-way ANOVA with Tukey test for multiple comparison (**p<0.01). Red stars indicate the genes expressed significantly higher in Mac-CF groups.

Because of the outstanding changes identified in the mitochondrial oxidative phosphorylation gene set, we further interrogated the enriched biological processes (BP) and molecular functions (MF) in Mac-CF MDM by Gene Ontology (GO) analysis. Due to the limited DEGs between Mac-CF and Ctrl MDM in Day 1 (**Supplementary Data 1**), the GO enrichment analysis was focused on Day 3 and 5 post-inoculation. Results (**Figures 4A, B**) indicate that DEGs of Mac-CF MDM enriched in the GO terms related to mitochondrial energy production, including ATP synthesis, electron transport, and oxidative phosphorylation, as compared to Ctrl MDM. Differences of these GO terms at Day 3 post-inoculation were more significant than those at Day 5 post-inoculation (**Figures 4A, B**). As the most enriched GO terms in Mac-CF MDM were related to NADH dehydrogenase activity (**Figure 4B**), we decided to quantify the gene scores for this activity (**Supplementary Data 2**). Mac-CF MDM had significantly higher scores for NADH dehydrogenase activity (GO:0003954) and NADH dehydrogenase (Quinone) activity (GO:0050136) at Day 3 post-inoculation (**Figures 4C, D**). Heatmap displays the most up- and down-regulated genes in the two gene sets (**Figure 4E**).

### 3.7 AM have similar transcriptional changes as MDM in Mac-CF lungs

**Figure 5:**
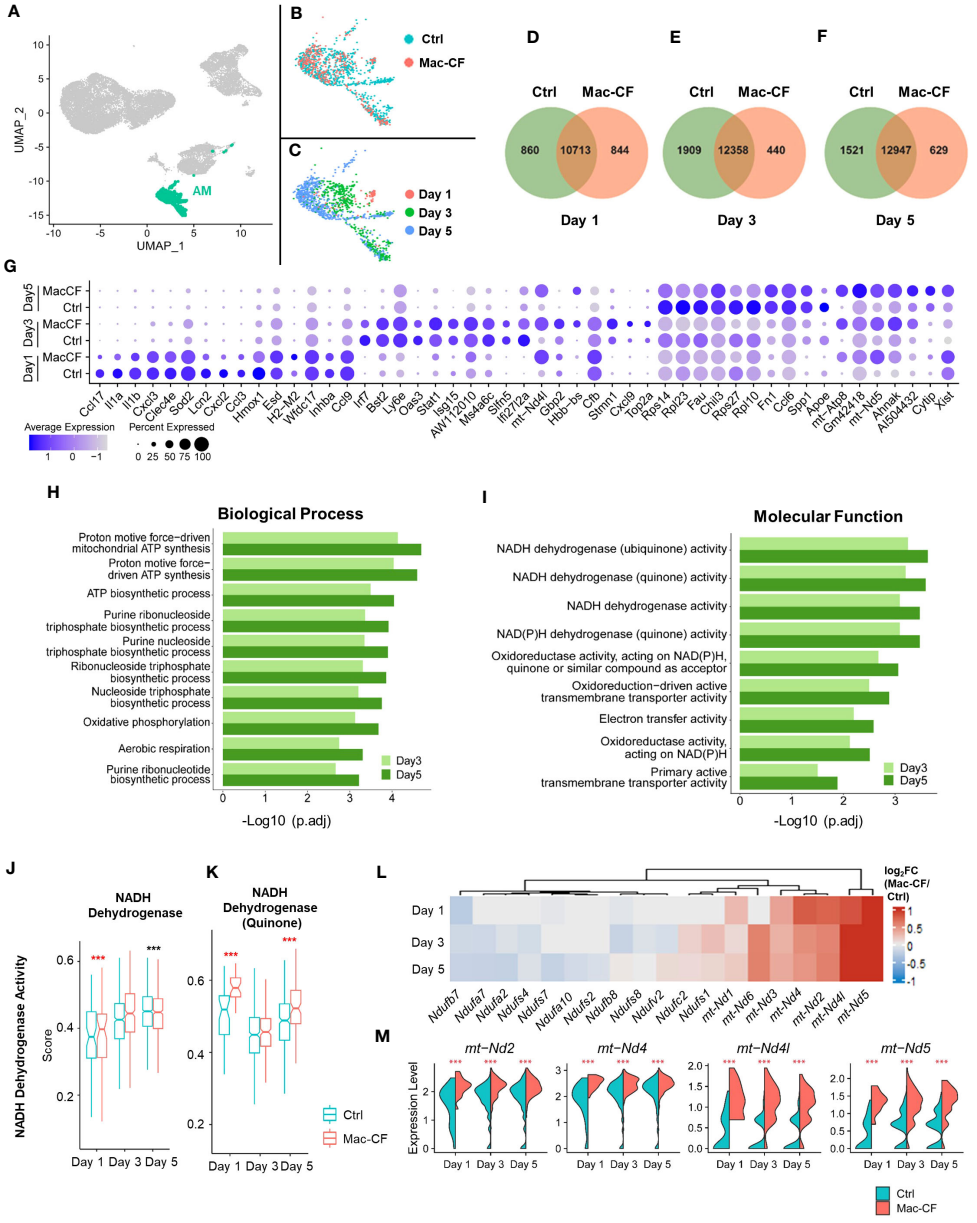
CF alveolar macrophages (AM) exhibit upregulation in mitochondrial respiration genes. **(A–C)** UMAP plots of the BALF AM from Ctrl and Mac-CF mice. **(A)** AM projection in all BALF cells; **(B)** AM projection by genotypes; **(C)** AM projection by time points. **(D–F)** Venn diagram plots indicating numbers of unique and overlapping genes (average gene counts >1) between Ctrl and Mac-CF AM cells at different time points (Day 1, 3, and 5 post inoculation). **(G)** Dot plot showing top 10 DEGs in the Ctrl and Mac-CF AM at Day 1, 3 and 5 post-inoculation. **(H, I)** GO enrichment of DEGs in Mac-CF AM as compared with Ctrl AM at Day 3 and 5 post-inoculation. **(H)** Top 10 GO biological process terms; **(I)** Top 10 GO molecular function terms. **(J, K)** Scores of pathways related to NADH dehydrogenase activity. **(J)** NADH dehydrogenase activity (GO:0003954); **(K)** NADH dehydrogenase (Quinone) activity (GO:0050136). **(L)** Heatmap showing the differences of log_2_[fold-change] in the expression of NADH dehydrogenase genes between Mac-CF and Ctrl AM. **(M)** Violin plots of expression level of representative genes associated with NADH dehydrogenase. Each p-value was assessed by two-way ANOVA with Tukey test for multiple comparison (***p<0.001). Red stars indicate the genes expressed significantly higher in Mac-CF groups compared to Ctrl groups, while black stars indicate the opposite.

Previous publications have documented that the Cx3cr1 promoter drives recombinase expression in not only MDM but also AM (33). We speculated that Mac-CF AM might share similar gene expression features as Mac-CF MDM. AM in the general UMAP of all BALF samples showed a stretched and branched pattern (**Figures 5A–C**). When plotted by genotypes, the projection patterns partially overlapped (**Figure 5B**). However, when plotted by time points, there was little to no overlapping (**Figure 5C**). Venn diagrams indicated that Mac-CF and Ctrl AM had expressed many of the same genes and a number of unique ones to each genotype at each of the three time points (**Figures 5D–F**). Similar to Mac-CF MDM, DEGs of Mac-CF AM were found to relate to mitochondrial function at Day 3 and 5 post-inoculation (**Figure 5G**). GO enrichment of DEGs from Mac-CF AM revealed the commonality between Mac-CF AM and Mac-CF MDM in upregulation of genes related to mitochondrial energy production and oxidative phosphorylation (**Figures 5H, I**; **Supplementary Data S2**). The scores of NADH dehydrogenase activity (GO:0003954) and NADH dehydrogenase (Quinone) activity (GO:0050136) revealed upregulation at Day 1 post-inoculation in Mac-CF AM (**Figures 5J, K**; **Supplementary Data S3**). Interestingly, the NADH dehydrogenase (Quinone) activity was up-regulated at Day 5 post-inoculation, while the NADH dehydrogenase activity was down-regulated (**Figures 5J, K**). Heatmap and violin plots display the upregulated genes from the two GO terms that may contribute to NADH dehydrogenase activity in Mac-CF AM (**Figures 5L, M**).

From the above data, we conclude that Mac-CF macrophages (MDM and AM) showed an altered transcriptional program, leading to significant upregulations in genes related to inflammatory response, immunity and mitochondrial respiration.

### 3.8 Loss of CFTR in macrophages alters the cell antioxidant system

**Figure 6:**
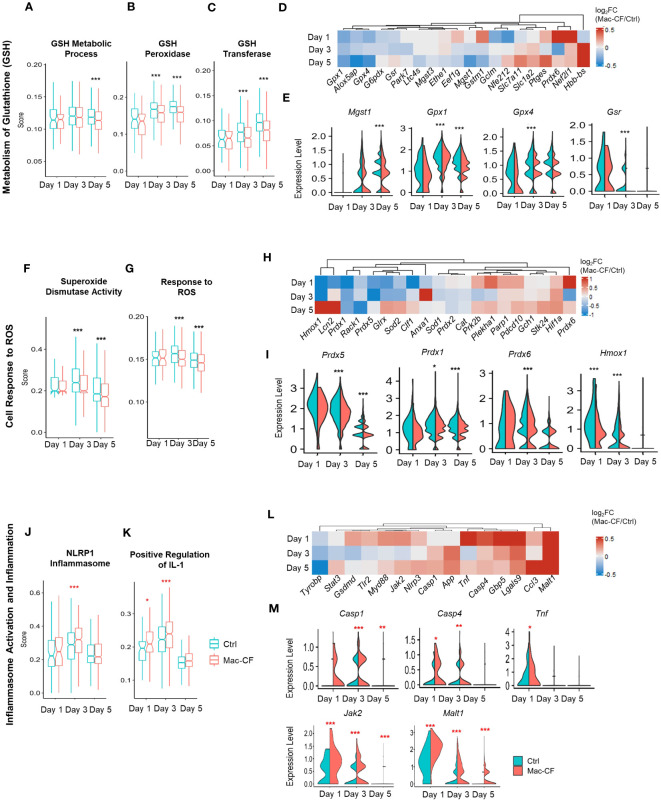
CF MDM exhibit loss in gene expression in pathways associated with antioxidant response and gain in inflammasome activity. **(A–E)** Scores of metabolism of glutathione (GSH) in Mac-CF MDM and Ctrl MDM. **(A)** Glutathione metabolic process (GO:0006749); **(B)** Glutathione peroxidase activity (GO:0004602); **(C)** Glutathione transferase activity (GO:0004364); **(D)** Heatmap showing the differences of log_2_[fold-change] in the expression of GSH metabolism related genes between Mac-CF and Ctrl MDM; **(E)** Violin plots of selected genes involved in the metabolism of GSH. **(F–I)** Scores of cell response to reactive oxygen species (ROS). **(F)** Superoxide dismutase activity (GO:0004784); **(G)** Cellular response to ROS (GO:0000302); **(H)** Heatmap showing the differences of log_2_[fold-change] in the expression of genes related to ROS response between Mac-CF and Ctrl MDM; **(I)** Violin plots of selected genes for the cell response to ROS. **(J–M)** Scores of inflammasome activation and inflammation. **(J)** NRLP1 inflammasome complex (GO:0072558); **(K)** Positive regulation of interleukin 1 production (GO:0032732); **(L)** Heatmap showing the differences of log_2_[fold-change] in the expression of genes related to inflammasome activation and inflammation between Mac-CF and Ctrl MDM; **(M)** Violin plots of selected gene expression levels involved in inflammasome activation and inflammation. Each p-value was assessed by two-way ANOVA with Tukey test for multiple comparison (*p<0.05, **p<0.01, ***p<0.001). Red stars indicate the genes expressed significantly higher in Mac-CF groups compared to Ctrl groups, while black stars indicate the opposite.

Complex 1 is the first of the five mitochondrial complexes, and the largest subunit in the electron transport chain for the generation of ATP. Oxidation of the NADH subunits (Nd1-Nd6) is the first step toward the transfer of electrons down to the other 4 complexes (67–69). Additionally, the activity of NADH dehydrogenase is linked to production of reactive oxygen species (ROS) (67, 68, 70). Due to the observed enhancement of NADH dehydrogenase gene activity, we reasoned that Mac-CF macrophages must be subject to an increased risk of ROS-induced damage. Thus, we decided to scrutinize the cell antioxidant pathways. As utilization of glutathione (GSH) is critical to removal of ROS (71–73), we first assessed the scores of GSH metabolism and the involving enzymes glutathione reductase (*Gsr*) and glutathione peroxidases (*Gpx*). Strikingly, the GSH metabolic process (GO:0006749) score was significantly lower in Mac-CF MDM than in Ctrl MDM at Day 5 post-challenge. Also, scores for GSH peroxidase activity (GO:0004602) and GSH transferase activity (GO:0004364) were significantly lower in Mac-CF MDM at Day 3 and 5 post-challenge (**Figures 6A–C**). Heatmap and violin plots reveal that Mac-CF MDM had significantly less expression of *Gpx1* and *Gsr* at Day 3 post-challenge, and *Gpx1* and *Mgst1* at Day 5 post-challenge (**Figures 6D, E**).

The superoxide dismutase (SOD) pathway represents another mechanism to scavenge the superoxide anion to mitigate ROS damage by converting superoxide into hydrogen peroxide for removal (71, 74). Our Mac-CF MDM had a significantly lower score of SOD activity (GO:0004784) or cellular response to ROS (GO:0000302) at Day 3 and 5 post-inoculation (**Figures 6F, G**). Mac-CF MDM exhibited down-regulation of *Prdx1*, *5*, and *6* that encode peroxiredoxins for the metabolism of hydrogen peroxide, and down-regulation of the Heme oxygenase-1 gene *Hmox1* (**Figures 6H, I**). HMOX expression has been previously found to be important in response to ROS and ROS-induced DNA damage (75, 76). Peroxiredoxins are known to be important for the metabolism of hydrogen peroxide (77, 78). Therefore, CFTR loss of function in MDM may undercut the cell antioxidant capacity. Our results are consistent with a previous publication reporting that Hmox1 expression and heme oxygenase 1 (HO-1) production were found to be reduced in CF human and mouse macrophages (79). It was further noted that a inducing Hmox1 expression in mice post-LPS exposure resulted in decreased lung neutrophil recruitment (79).

Varying from Mac-CF MDM, Mac-CF AM had no difference in GSH metabolism score, as compared to Ctrl AM. However, like Mac-CF MDM, Mac-CF AM also exhibited significantly lower scores for GSH peroxidase and GSH transferase activity at Day 3 and 5 post-inoculation (**Supplementary Figures 3B, C**; **Supplementary Data 3**). The differences of log_2_[fold-change] in genes associated with GSH metabolism process revealed varying expression between the Mac-CF AM cells and the Ctrl AM cells (**Supplementary Figure 3D**). Furthermore, Mac-CF AM increased *Mgst1* expression at Day 1 post-inoculation and reduced *Gpx1* expression at Day 5 post-inoculation. *Gpx4* expression was decreased in Mac-CF AM cells at Day 3 and 5 post-inoculation (**Supplementary Figure 3E**). Unlike Mac-CF MDM, there were no differences in scores for SOD pathway and cellular response to ROS in Mac-CF AM (**Supplementary Figures 3F, G**). Heatmap shows the differences of log_2_[fold-change] between Mac-CF and Ctrl groups in expression of the genes in this pathway (**Supplementary Figure 3H**). Specifically, Mac-CF AM had increased expression of *Prdx5* at Day 5 post-inoculation, and *Sod2* at Day 3 post-inoculation. Mac-CF AM cells had no difference in the expressions of *Prdx1* and *Hmox1* (**Supplementary Figure 3I**). Therefore, MDM may be more transcriptionally affected by the loss of CFTR than AM in our Mac-CF mouse lungs.

### 3.9 Loss of CFTR in macrophages enhances the inflammasome pathway

Formation of inflammasome is a critical event for the release of inflammatory cytokines. ROS produced by mitochondria have been correlated with an increase in inflammasome assembly in CF (80–82). Therefore, we investigated if Mac-CF MDM had upregulated expression of genes associated with inflammasome formation. As shown, Mac-CF MDM revealed higher scores for NRLP1 inflammasome complex (GO:0072558) at Day 3 post-inoculation, and for positive regulation of IL-1 production (GO:0032732) at Day 1 and 5 post-inoculation (**Figures 6J, K**). Additionally, examination of the two gene sets revealed upregulation of multiple proinflammatory genes (**Figure 6L**), such as *Casp1*, *Casp4*, *Tnf*, *Jak2*, and *Malt1* (**Figure 6M**).

In contrast to Mac-CF MDM, Mac-CF AM did not display any similar upregulation in the NLRP1 inflammasome complex score (**Supplementary Figure 3J**) and the score of positive regulation of IL-1 production(**Supplementary Figure 3K**). Unlike Mac-CF MDM, Mac-CF AM had no significant upregulation in the expressions of *Casp1*, *Casp4*, *Jak2*, and *Malt1*, and the Ctrl AM cells had increased expression of *Tnf* only at Day 1 post-inoculation (**Supplementary Figures 3L, M**). These data indicate that Mac-CF MDM and AM may differ in their inflammasome response to PsA challenge. Thus, Mac-CF MDM cells may contribute relatively more to the lung pro-inflammatory status than Mac-CF AM.

## 4 Discussion

Dysregulated inflammation in CF lungs is one of the major factors that lead to lung damage. Newly implemented highly effective CFTR modulator therapies can effectively correct CF epithelial defects, reflected by normalization of the sweat Cl^–^ transport (83, 84). However, lung inflammation persists after initial reduction (85). As CF lung disease is commonly accepted as a disease of epithelial dysfunction, how CF immune cells are involved in the disease pathogenesis is not fully understood. Utilizing the Mac-CF mouse model, we investigated the direct contribution of CF macrophages to lung infection and inflammation. The results demonstrated that CFTR loss of function in macrophages alone does not affect lung bacterial clearance, but delays lung resolution of inflammation.

It was a surprise that the Mac-CF lungs cleared the inoculated bacteria as effectively as the Ctrl lungs. Previous studies have demonstrated that macrophages isolated from CF patients kill bacteria less efficiently than non-CF macrophages *in vitro* (86–88). Our current data also showed that BMDM from Mac-CF mice killed PsA less efficiently than Ctrl BMDM *in vitro* (**Supplementary Figure 2H**). This *in vivo* and *in vitro* data discrepancy can be explained by potential functional compensation by other immune cells *in vivo*. For example, in our Mac-CF mice, neutrophils, the major phagocytes in bacterial clearance, had intact *Cftr* and should be functionally normal.

CF lungs are marked by chronic inflammation with pronounced neutrophilic infiltration (89). We discovered in the current study that loss of CFTR in macrophages alone resulted in a prolonged inflammation after PsA challenge. At Day 3 post-inoculation, Mac-CF lungs contained significantly more neutrophils and produced significantly more proinflammatory cytokines/chemokines (IL-1β, TNF-α, IL-6, and KC) (**Figures 1K, M–Q**). Interestingly, by Day 5 post-inoculation, Mac-CF mice resolved the difference in inflammation (**Figures 1G–J**). Noteworthy is that at Day 2 post-inoculation, both lungs had a comparable level of inflammation, indicating a comparable inflammatory response at the early phase of infection. Thus, the observed difference in inflammation at Day 3 post-inoculation must arise from dysregulated resolution. As there was no difference in bacterial load between the Ctrl and Mac-CF lungs, the level of extracellular stimulation to macrophages should be similar. Thus, we reason that the extended hyper-inflammation in Mac-CF lungs must be due to prolonged intracellular stimulation and/or hyper-responsiveness of Mac-CF macrophages. In support of this reasoning, we found that BMDM from Mac-CF mice produced significantly more pro-inflammatory cytokines/chemokines under an identical level of LPS stimulation (**Supplementary Figures 2C–F**). Thus, CF macrophages have an intrinsic defect in immune response to bacterial challenge.

To understand molecularly how CFTR loss of function in macrophages leads to the intrinsic immune defect, we performed scRNA-seq on BALF cells obtained from Ctrl and Mac-CF mouse lungs after PsA challenge. The most significantly upregulated genes in Mac-CF MDM (**Figure 3H**) were related to not only cytokines/chemokines (*IL-1α*, *Tnf* and *Ccl6*) and immunity (*Malt1*, *Stat1*, *Ifi209*, *Slfn5*, *Gbp2* and *Ifit2*), but also mitochondrial oxidative phosphorylation (*mt-ND2, 4, 4L, 5, mt-Co1* and *mt-Atp6*). More importantly, GO enrichment analysis revealed that most DEGs in Mac-CF macrophages were enriched in mitochondrial oxidative phosphorylation, aerobic respiration, ATP biosynthesis, and oxidoreductase activity (**Figures 4A, B**; **Supplementary Data 2**). Strikingly, multiple mitochondrial NADP dehydrogenase subunit genes were among the top-listed DEGs. Many publications have documented the correlation of aberrant mitochondrial function in many diseases, including CF, with excessive production of ROS and inflammatory cytokines (90–92). Thus, Mac-CF macrophages, having a dysregulated mitochondrial function, may facilitate their hyper-inflammation propensity.

Converting ROS into non-harmful molecules is critical to protection of tissues from ROS-induced damage (71, 93). GSH, a tripeptide directly transported by the CFTR channel, plays an important role in the ROS-induced stress response (94, 95). Indeed, both CF lung and CF cells have lower levels of GSH, correlated with increased ROS production (96, 97). It was further discovered that a decrease in intracellular mitochondrial GSH results in an inflammatory state, marked by an increase in inflammatory cytokines and ROS production (96–98). Moreover, CF macrophages exhibited a loss of GSH, resulted in increased ROS and TGF-β production (98). Our scRNA-seq data indicated that GSH metabolism score was significantly lower in Mac-CF MDM than in Ctrl MDM at Day 3 post-challenge, and scores for GSH peroxidase and GSH transferase were significantly lower in Mac-CF MDM at Day 3 and 5 post-inoculation (**Figures 6A–C**). This sub-optimal antioxidant state may contribute to more severe tissue damage in CF lungs.

Inflammasome formation has been connected to ROS production and subsequent release of inflammatory cytokines (99–101). Activation of inflammasomes is known to result in activation of caspase-1 and subsequent maturation of pro-IL-1β and pro-IL-18 to IL-1β and IL-18 (99, 102, 103). Indeed, human macrophages exhibited an increased formation of the NRLP3 inflammasome following exposure to PsA (104). Isolated monocytes from CF patients also exhibited increased NLRP3 inflammasome activity following stimulation with LPS (105). The inflammasome can also be activated through a non-canonical pathway through sensing of internalized LPS, leading to the activation of caspase 4, also referred to as caspase 11, for the release of IL-1β, IFN, and NFκ-B (106–108). Our current data have demonstrated that Mac-CF MDM had a significantly higher score of inflammasome formation and significantly higher expression levels of *Casp1* at Day 3 post-inoculation and *Casp4* at Day 1 and 3 post-inoculation (**Figures 6J–M**). These data suggest that Mac-CF macrophages have activated inflammasome pathways, which may explain why more IL-1β was observed in Mac-CF lungs. Studies using clinical CF patients’ monocytes proved that CFTR modulator therapy decreased levels of markers of inflammasome formation and IL-1β production (109, 110).

It is noteworthy that CX3CR1-Cre can target more than one macrophage population (33), including alveolar macrophages, monocyte-derived macrophages, and dendritic cells. We thus believe that *Cftr* would be deleted in all these cells. However, our scRNA-seq data clearly indicated that in the infected and inflamed lungs, monocyte-derived macrophages were the predominant macrophage type 3 days post inoculation, thus affecting the lung pathogenesis.

In summary, this research has provided comprehensive data to suggest that CFTR loss of function in macrophages delays lung resolution of inflammation. Macrophages from Mac-CF lungs have an aberrant transcriptional program that confers the cells with a hyper-inflammatory propensity, thus facilitating a greater lung inflammation and a slow resolution process.

## Data availability statement

The datasets presented in this study can be found in online repositories. The names of the repository/repositories and accession number(s) can be found below: GSE233733 (GEO).

## Ethics statement

The animal study was approved by Louisiana State University Health Sciences Center Animal Care and Use Committee. The study was conducted in accordance with the local legislation and institutional requirements.

## Author contributions

DW performed the experiments and data analyses. YH led and performed the scRNA-seq data analyses. SJ was involved in animal model establishment and colony maintenance. GW conceived and designed the experiments and secured the funding. DW drafted, YH and GW revised, and SJ proofread the manuscript. All authors contributed to the article and approved the submitted version.
