# Supplementary material for: Loss of CFTR function in macrophages alters the cell transcriptional program and delays lung resolution of inflammation
Source: Front Immunol. 2023 Nov 16;14:1242381. doi: 10.3389/fimmu.2023.1242381 (PMC10687418; doi:10.3389/fimmu.2023.1242381)
Supplement: Supplementary file 1 [file DataSheet_1.pdf]

frontiers

Supplementary Material

Loss of CFTR function in macrophages alters the cell transcriptional program and delays lung resolution of inflammation

Dianne Wellems, Yawen Hu, Scott Jennings, Guoshun Wang*

*** Correspondence:** Guoshun Wang: gwang@lsuhsc.edu

1 Supplementary Data

1.1 Supplementary Data 1: Differentially Expressed Genes (DEGs) in MDM and AM (Mac-CF vs Ctrl)

1.2 Supplementary Data 2: Gene Ontology (GO) of MDM and AM (Mac-CF vs Ctrl)

1.3 Supplementary Data 3: Log_2fold Gene Expression Differences (Mac-CF vs Ctrl)

2 Supplementary Tables and Figures

2.1 Supplementary Table 1

Reagent	Source	Identifier
Antibodies		
Rat Anti-Mouse FITC F4/80, Clone BM8	BioLegend	Cat#123108, RRID:AB_893502
Rat FITC IgG Isotype, Clone BR2a	Invitrogen eBiosciences	Cat#11-4321-80, RRID:AB_1834375
Bacterial Strain		
Pseudomonas aeruginosa (clinical isolate)	Dr. Paul D. Fey, UNMC	N/A
Chemicals		
TruStain FcX^{TM} PLUS (anti-mouse CD16/32)	Biolegend	Cat#156604 RRID: 156604
Tryptic Soy Broth (TSB)	Sigma	Cat#22092
PBS (-Mg, -Cl)	Gibco	Cat#14190144
AOPI Staining Solution	Nexcelom ViaStain	Cat#CS2-0106
Harleco Hemacolor Stain Kit	Sigma-Aldrich	Cat#65092-93
Dulbecco's Modified Eagle Medium (DMEM)	Gibco	Cat#11965-092
GlutaMAX	ThermoFisher	Cat#35050061
Antibiotic-Antimycotic	Gibco	Cat#15240096
Human AB Serum	Sigma	Cat#HS3667
Pseudomonas aeruginosa Lipopolysaccharide (LPS)	Sigma	Cat#L9143
Gentamicin	Sigma	Cat#G1914
Saponin	Sigma	Cat#47036-50G-F
Commercial Assays		
Mouse DuoSet ELISA IL-6	R&D systems	Cat#DY-406
Mouse DuoSet ELISA TNF-α	R&D systems	Cat#DY-410
Mouse DuoSet ELISA MIP-2	R&D systems	Cat#DY-452
Mouse DuoSet ELISA IL-1β	R&D systems	Cat#DY-401
Mouse DuoSet ELISA KC	R&D systems	Cat#DY-453
Cell Lines		
Mouse Fibroblast, L929 cell	ATCC	RRID: CVCL_0462
Animal Lines		
Cftr$^{fl10/fl10}$ mouse line	(1)	N/A
B6J.B6N(Cg)-Cx3cr1tm1.1(cre)Jung/J mouse line	(2)	RRID: IMSR_JAX:025524
Macrophage-*Cftr*-exon-10-KO (Mac-CF) mouse line	In House, LSUHSC	N/A
Software and Packages		
GraphPad Prism version 9.5.1 (733)	GraphPad Software	https://www.graphpad.com/features

FlowJo version 10.8	BD	https://www.flowjo.com/solutions/flowjo
R version 4.2.2 and R Studio Open Source version 2022.12.0 Build 353	(3-8)	https://posit.co/download/rstudio-desktop/S
Seurat Package Version 4.30	(4-7)	https://satijalab.org/seurat/articles/install.html
glmGamPoi version 1.10.2	(9, 10)	N/A
Immunological Genome Project (ImmGen) GRCm38 release M25	(11, 12)	N/A
SingleR version 2.0.0	(12)	N/A
ClusterProfiler version 4.6.0	(13, 14)	N/A
Mouse Database (org.Mm.eg.db) version 3.16.0	(15)	https://bioconductor.org/packages/release/data/annotation/html/org.Mm.eg.db.html
BiocManager version 1.30.20	(16)	https://www.bioconductor.org/install/
GSEA Database	(17-20)	N/A
Gene Ontology Terms		
GO Molecular Function (GOMF) NADH dehydrogenase activity	GO:0003954	https://www.gsea-msigdb.org/gsea/msigdb/mouse/geneset/GOMF_NADH_DEHYDROGENASE_ACTIVITY.html
GOMF NADH dehydrogenase (quinone) activity	GO:0050136	https://www.gsea-msigdb.org/gsea/msigdb/no_such_gene_set.jsp?targetSpeciesDB=Mouse&geneSetName=GOMF_NADH_DEHYDROGENASE_QUINONE_ACTIVITY
GO Biological Process (GOBP) Glutathione metabolic processes	GO:0006749	https://www.gsea-msigdb.org/gsea/msigdb/mouse/geneset/GOBP_GLUTATHIONE_METABOLIC_PROCESS.html
GOMF Glutathione peroxidase activity	GO:0004602	https://www.gsea-msigdb.org/gsea/msigdb/mouse/geneset/GOMF_GLUTATHIONE_PEROXIDASE_ACTIVITY.html
GOMF Glutathione transferase activity	GO:0004364	https://www.gsea-msigdb.org/gsea/msigdb/mouse/geneset/GOMF_GLUTATHIONE_TRANSFERASE_ACTIVITY.html
GO Cellular Component (GOCC) NRLP1 inflammasome complex	GO:0072558	https://www.gsea-msigdb.org/gsea/msigdb/m

		ouse/geneset/GOCC_NLRP1_INFLAMMASOME_COMPLEX.html
GOBP Positive regulation of interleukin 1 production	GO:0032732	https://www.gsea-msigdb.org/gsea/msigdb/mouse/geneset/GOBP_POSITIVE_REGULATION_OF_INTERLEUKIN_1_PRODUCTION.html
GOMF Superoxide dismutase activity	GO:0004784	https://www.gsea-msigdb.org/gsea/msigdb/mouse/geneset/GOMF_SUPEROXIDE_DISMUTASE_ACTIVITY.html
GOBP Response to reactive oxygen species	GO:0000302	https://www.gsea-msigdb.org/gsea/msigdb/mouse/geneset/GOBP_RESPONSE_TO_REACTIVE_OXYGEN_SPECIES.html
Other		
20G blunt cannula	Covidien Monoject	Cat#8881202363
Cellometer K2 PD3000	Nexcelom	N/A
70-micron strainer	Greiner Bio-One	Cat#542170

2.2 Supplementary Figure 1

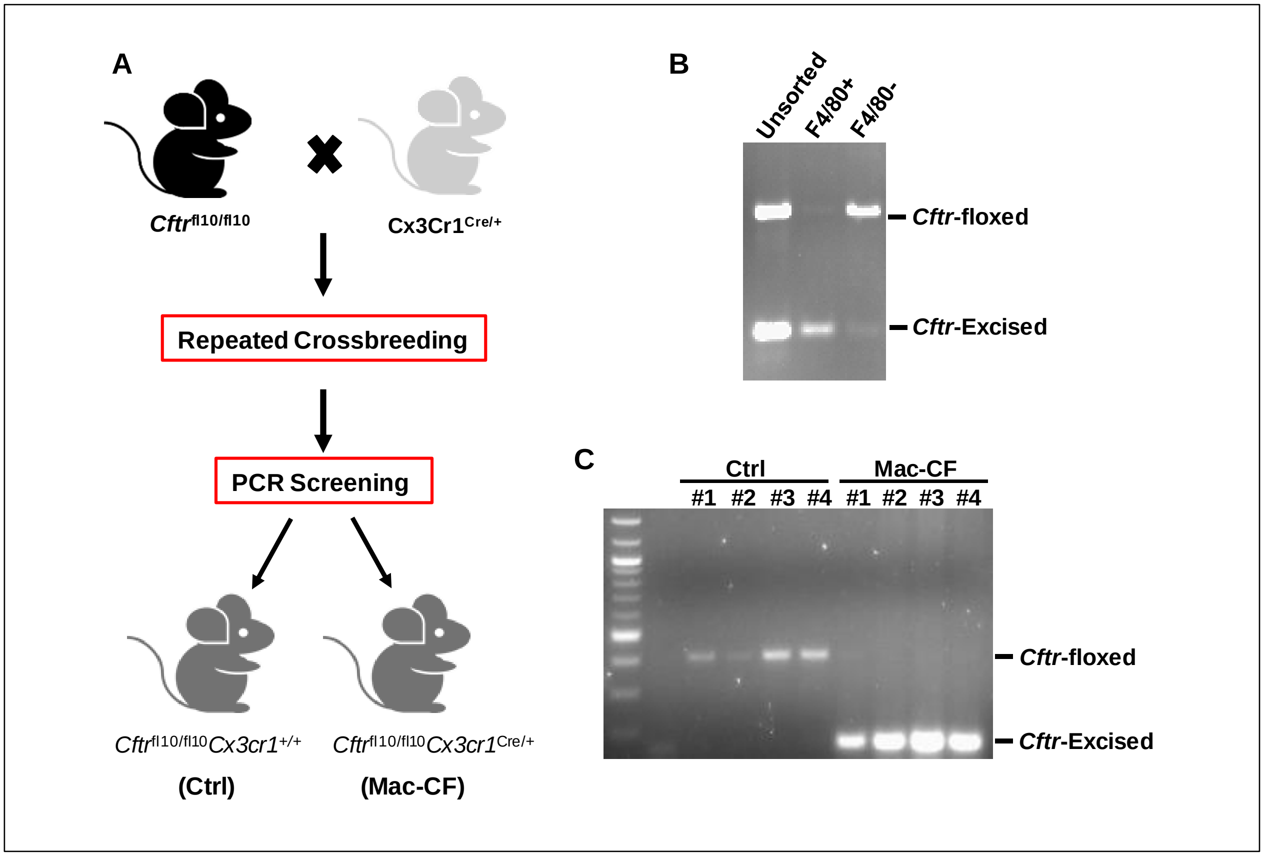

Supplementary Figure 1: Generation and validation of macrophage-specific CF (Mac-CF) mouse model

(A) Schematic of generation of Mac-CF mouse model. *Cftr*$^{fl10/fl10}$ mice were bred with Cx3Cr1-Cre mice. After repeated crossbreeding and PCR screening, Mac-CF mice (*Cftr*$^{fl10/fl10}$*Cx3cr1*$^{cre/+}$) and congenic control (Ctrl) mice (*Cftr*$^{fl10/fl10}$*Cx3cr1*$^{+/+}$) were obtained.

(B) Validation of macrophage-specific *Cftr*-exon-10 deletion. Peritoneal inflammatory cells, induced by casein injection, were collected and FACS-sorted for F4/80-positive or negative cells. Unsorted cells show both *Cftr*-floxed and excised bands, while F4/80-positive cells only have the excised band, and F4/80-negative cells the floxed band.

(C) Bone marrow-derived macrophages have *Cftr*-exon-10 deletion. Macrophages, differentiated in culture from the bone marrow cells of Ctrl and Mac-CF mice (4 mice each), were PCR genotyped for *Cftr*-exon-10 deletion. Ctrl cells have the *Cftr*-floxed band, and Mac-CF cells show the excised band.

2.3 Supplementary Figure 2

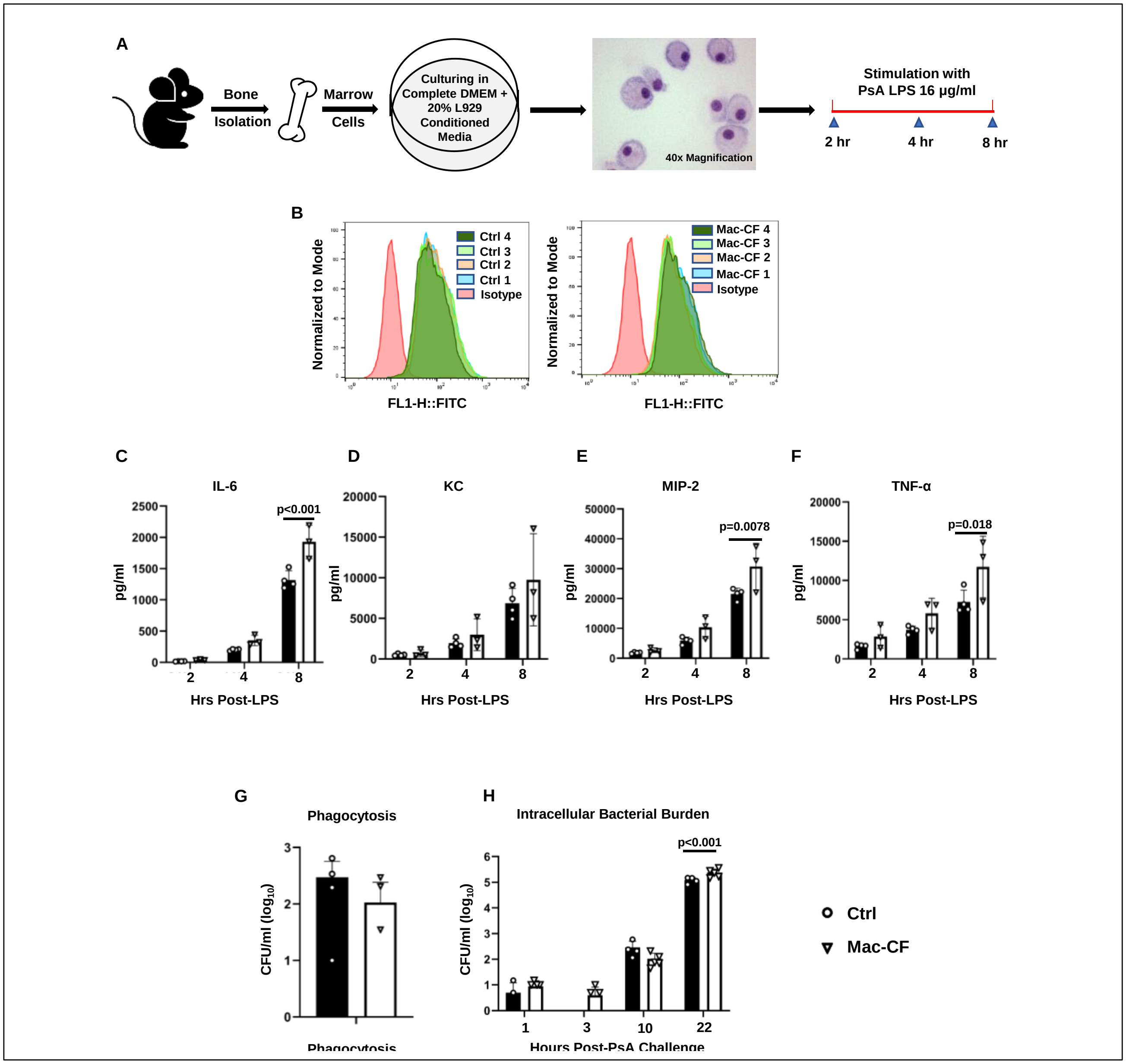

Supplementary Figure 2: Loss of CFTR in macrophages derived from Mac-CF bone marrow results in over-production of inflammatory cytokines/chemokines, and increased bacterial burden *in vitro*

(A) Schematic for bone marrow isolation and bone marrow derived macrophage (BMDM) differentiation.

(B) Immune staining and flow cytometry to validate macrophage differentiation. FITC-F4/80 antibody staining and FITC-IgG rat isotype antibody staining demonstrate a total population shift of the differentiated macrophages from 4 mice per genotype.

(C-F) Inflammatory cytokine levels in the supernatants of BMDM post-LPS stimulation (16 µg/ml). Data represents 3-4 mice per genotype. Significance was calculated using two-way ANOVA with Tukey test.

(G-H) In vitro bacterial killing. BMDM were exposed with human AB serum-opsonized *Pseudomonas aeruginosa* at 6 MOI. (G) Phagocytosis 30 minutes post-exposure. (H) Intracellular bacterial survival. Data represent 4-5 mice per genotype. Significance was calculated using two-way ANOVA with Tukey test.

2.4 Supplementary Figure 3

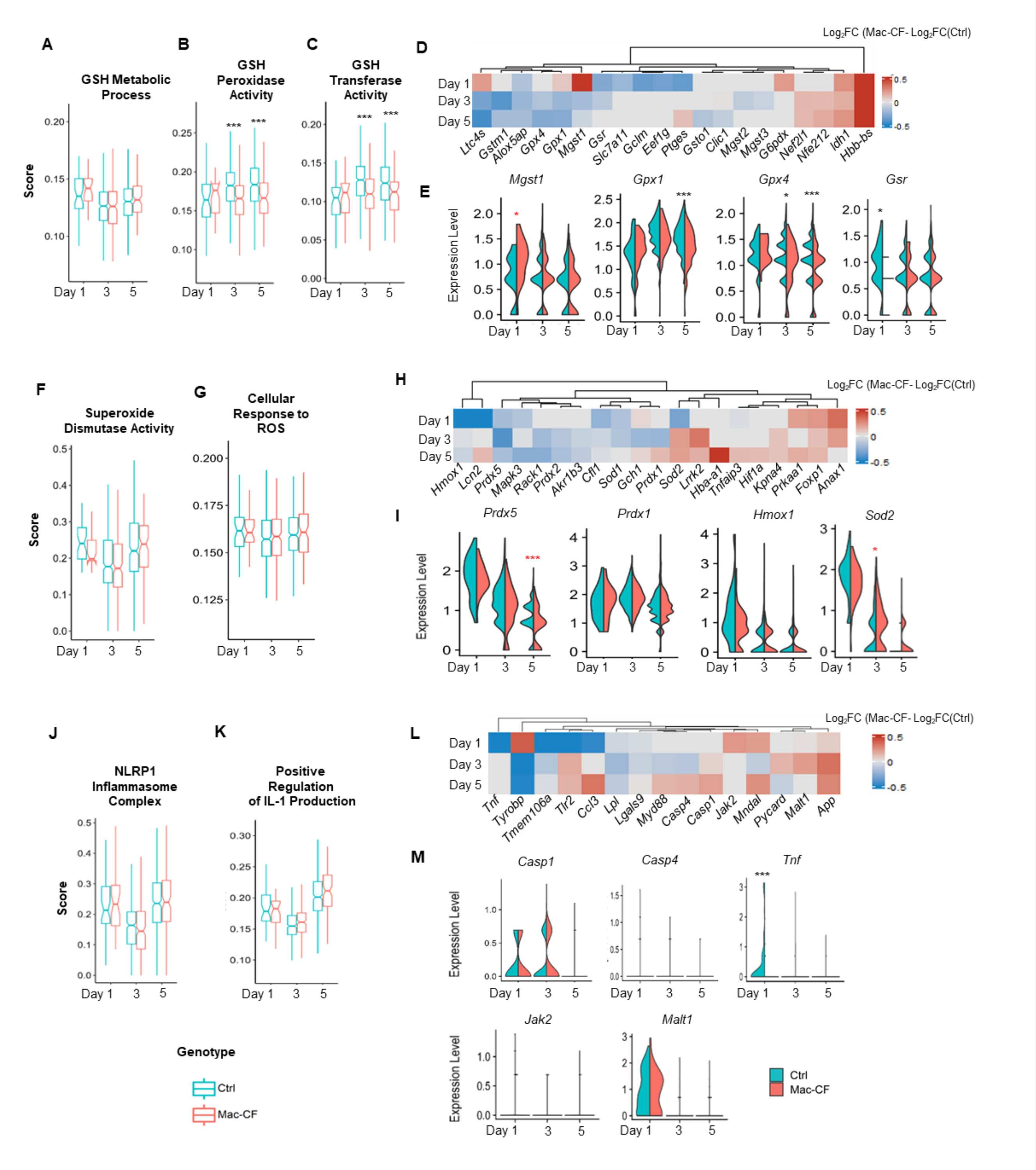

Supplementary Figure 3: Pathway analysis of Ctrl and Mac-CF AM

(A-E) Scores of metabolism of glutathione (GSH) of Mac-CF alveolar macrophages (AM) as compared to Ctrl AM. (A) Glutathione metabolic process (GO:0006749); (B) Glutathione peroxidase activity (GO:0004602); (C) Glutathione transferase activity (GO:0004364). (D) Heatmap showing the differences of $\log_2$[fold-change] in the expression of GSH metabolism-related genes between Mac-CF and Ctrl AM. (E) Violin plot showing the expression levels of selected genes involved in the metabolism of GSH.

(F-I) GO enrichment in cellular response to ROS. (F) Superoxide dismutase activity (GO:0004784); (G) Cellular response to reactive oxygen species (GO:0000302). (H) Heatmap showing the differences of $\log_2$[fold-change] in the expression of genes related to ROS response. (I) Violin plots showing the expression levels of selected genes related to cell response to ROS.

(J-M) Scores of inflammasome activation and inflammation. (J) NRLP1 inflammasome complex (GO:0072558); (K) Positive regulation of interleukin 1 production (GO:0032732); (L) Heatmap showing the differences of $\log_2$[fold-change] of selected genes in Mac-CF AM as compared with Ctrl AM. (M) Violin plots for selected gene expressions involved in inflammasome activation and inflammation.

Each p-value was assessed by two-way ANOVA with Tukey test for multiple comparison (*$p<0.05$, **$p<0.01$, ***$p<0.001$). Red stars indicate that the genes are expressed significantly higher in Mac-CF AM, while black stars indicate the opposite. Data represent 4 mice per genotype and per time point.

3 References

1. Hodges CA, Cotton CU, Palmert MR, Drumm ML. Generation of a conditional null allele for Cftr in mice. Genesis. 2008;46(10):546-52.

2. Yona S, Kim KW, Wolf Y, Mildner A, Varol D, Breker M, et al. Fate mapping reveals origins and dynamics of monocytes and tissue macrophages under homeostasis. Immunity. 2013;38(1):79-91.

3. Team RC. R: A language and environment for statistical computing. 2013.

4. Hao Y, Hao S, Andersen-Nissen E, Mauck WM, 3rd, Zheng S, Butler A, et al. Integrated analysis of multimodal single-cell data. Cell. 2021;184(13):3573-87 e29.

5. Stuart T, Butler A, Hoffman P, Hafemeister C, Papalexi E, Mauck WM, 3rd, et al. Comprehensive Integration of Single-Cell Data. Cell. 2019;177(7):1888-902 e21.

6. Butler A, Hoffman P, Smibert P, Papalexi E, Satija R. Integrating single-cell transcriptomic data across different conditions, technologies, and species. Nat Biotechnol. 2018;36(5):411-20.

7. Satija R, Farrell JA, Gennert D, Schier AF, Regev A. Spatial reconstruction of single-cell gene expression data. Nat Biotechnol. 2015;33(5):495-502.

8. Team R. RStudio: integrated development for R. RStudio, PBC, Boston, MA. 2020. 2021.

9. Hafemeister C, Satija R. Normalization and variance stabilization of single-cell RNA-seq data using regularized negative binomial regression. Genome Biol. 2019;20(1):296.

10. Choudhary S, Satija R. Comparison and evaluation of statistical error models for scRNA-seq. Genome Biol. 2022;23(1):27.

11. Heng TS, Painter MW, Immunological Genome Project C. The Immunological Genome Project: networks of gene expression in immune cells. Nat Immunol. 2008;9(10):1091-4.

12. Aran D, Looney AP, Liu L, Wu E, Fong V, Hsu A, et al. Reference-based analysis of lung single-cell sequencing reveals a transitional profibrotic macrophage. Nat Immunol. 2019;20(2):163-72.

13. Wu T, Hu E, Xu S, Chen M, Guo P, Dai Z, et al. clusterProfiler 4.0: A universal enrichment tool for interpreting omics data. Innovation (Camb). 2021;2(3):100141.

14. Yu G, Wang LG, Han Y, He QY. clusterProfiler: an R package for comparing biological themes among gene clusters. Omics. 2012;16(5):284-7.

15. Carlson M. org. Mm. eg. db: Genome wide annotation for Mouse. R package version 3.8.2. Bioconductor London, United Kingdom: Genome Biology (BMC). 2019.

16. Morgan M. BiocManager: access the bioconductor project package repository. R package version 13020. 2023;1(10).

17. Subramanian A, Tamayo P, Mootha VK, Mukherjee S, Ebert BL, Gillette MA, et al. Gene set enrichment analysis: a knowledge-based approach for interpreting genome-wide expression profiles. Proc Natl Acad Sci U S A. 2005;102(43):15545-50.

18. Liberzon A, Birger C, Thorvaldsdottir H, Ghandi M, Mesirov JP, Tamayo P. The Molecular Signatures Database (MSigDB) hallmark gene set collection. Cell Syst. 2015;1(6):417-25.

19. Liberzon A, Subramanian A, Pinchback R, Thorvaldsdottir H, Tamayo P, Mesirov JP. Molecular signatures database (MSigDB) 3.0. Bioinformatics. 2011;27(12):1739-40.

20. Mootha VK, Lindgren CM, Eriksson KF, Subramanian A, Sihag S, Lehar J, et al. PGC-1alpha-responsive genes involved in oxidative phosphorylation are coordinately downregulated in human diabetes. Nat Genet. 2003;34(3):267-73.
